# Odorous Compounds from Poultry Manure Induce DNA Damage, Nuclear Changes, and Decrease Cell Membrane Integrity in Chicken Liver Hepatocellular Carcinoma Cells

**DOI:** 10.3390/ijerph14080933

**Published:** 2017-08-18

**Authors:** Adriana Nowak, Tadeusz Bakuła, Katarzyna Matusiak, Remigiusz Gałęcki, Sebastian Borowski, Beata Gutarowska

**Affiliations:** 1 Institute of Fermentation Technology and Microbiology, Lodz University of Technology, Wolczanska 171/173, 90-924 Lodz, Poland; katarzyna.matusiak@p.lodz.pl (K.M.); sebastian.borowski@p.lodz.pl (S.B.); beata.gutarowska@p.lodz.pl (B.G.); 2 Department of Veterinary Prevention and Feed Hygiene, University of Warmia and Mazury in Olsztyn, Oczapowskiego 13, 10-718 Olsztyn, Poland; bakta@uwm.edu.pl; 3 Faculty of Veterinary Medicine, University of Warmia and Mazury, ul. Oczapowskiego 13, 10-719 Olsztyn, Poland; remigiusz.galecki@gmail.com

**Keywords:** poultry odours, genotoxicity, cytotoxicity, DNA damage, comet assay, LDH assay, DAPI staining

## Abstract

Animal breeding and management of organic wastes pose a serious problem to the health of livestock and workers, as well as the nearby residents. The aim of the present study was to determine the mechanisms of toxicity of selected common odorous compounds from poultry manure, including ammonia, dimethylamine (DMA), trimethylamine (TMA), butyric acid, phenol, and indole. We measured their genotoxic and cytotoxic activity in the model chicken cell line (LMH), in vitro, by comet assay and lactate dehydrogenase assay, respectively. We also made microscopic observations of any morphological changes in these cells by DAPI staining. Four compounds, namely ammonia, DMA, TMA, and butyric acid increased DNA damage in a dose-dependent manner (*p* < 0.05), reaching genotoxicity as high as 73.2 ± 1.9%. Phenol and indole induced extensive DNA damage independent of the concentration used. Ammonia, DMA, and TMA caused a dose-dependent release of lactate dehydrogenase (*p* < 0.05). The IC_50_ values were 0.02%, 0.05%, and 0.1% for DMA, ammonia and TMA, respectively. These compounds also induced nuclear morphological changes, such as chromatin condensation, shrinkage, nuclear fragmentation (apoptotic bodies), and chromatin lysis. Our study exhibited the damaging effects of odorous compounds in chick LMH cell line.

## 1. Introduction

Air emission sources from animal production include buildings, animal feedlot surfaces, manure storage and treatment units, silage piles, and a variety of other smaller emission sources [1]. Emissions of odorous compounds from chicken sheds can lead to odour problems in the surrounding area, which lead to complaints from residents [2]. An unpleasant odour is associated with poultry manure, which results from a combination of up to 150 different compounds, including ammonia, amines, mercaptans, aldehydes, hydrogen sulphide, sulphur compounds, and esters [2,3,4]. The odours are mainly the products of the decomposition of chicken faeces, feathers, and litter by aerobic and anaerobic microorganisms. Each of these sources has a different emission profile, fluctuating during the day and throughout the year [1]. Thus, an effective odour removal technique is a great challenge for researchers. In our previous studies, we demonstrated that the treatment of poultry manure with mineral-microbial preparation successfully reduced odorants from feedstock. The odorants included ammonia, di- and trimethylamine, isobutyric acid, and other odorous compounds. We developed a novel microbial-mineral preparation composed of perlite and bentonite with spray-dried microorganisms, which was selected on the basis of active removal of odours from poultry manure [5,6].

Ammonia, dimethylamine (DMA), trimethylamine (TMA), indole, phenol, and butyric acid are the most common compounds present in poultry manure [7,8,9]. Their health effects on humans and animals were discussed, in depth, in our previous study [10]. In summary, odorous compounds with long-term exposure can cause irritation of mucosal membranes in the respiratory tract in farm chickens, tracheal irritation, air sac inflammation, conjunctivitis, dyspnoea, respiratory tract damage, reddening, corneal clouding, reduction in respiratory rate, and central nervous system disturbances [10]. There is still insufficient in vitro data associated with the cyto- and genotoxicity of poultry odorous compounds with the application of cell lines. Thus far, we demonstrated the cytotoxicity of selected odorous compounds using the MTT (3-(4,5-dimethylthiazol-2-yl)-2,5-diphenyltetrazolium bromide) and PrestoBlue assays in the chicken Leghorn Male Hepatoma (LMH) cell line [10]. This helped us select appropriate concentrations of the odorous compounds for genotoxicity testing for this study. Here, we wanted to investigate the mechanisms of toxicity of selected odorous compounds, and examine if cytotoxicity is accompanied by genotoxicity, which can lead to mutations and cancer.

The aim of this study was to measure the genotoxicity (measurement of DNA damage using the comet assay) and cytotoxicity (necrosis in lactate dehydrogenase assay, along with IC_50_ values) of the most common odorous compounds from poultry manure in the chick LMH cell line, in vitro. In addition, we microscopically examined any morphological changes in the cellular nuclei (apoptosis or necrosis after fluorescent staining).

## 2. Materials and Methods

### 2.1. Chemicals

Ammonia, DMA, TMA, indole, phenol, and butyric acid were purchased from Sigma-Aldrich, St. Louis, MO, USA. The stocks were dissolved in Waymouth’s Medium (Gibco, Thermo Fisher Scientific, Waltham, MA, USA) with no supplements and filter sterilized (0.22 μM pore size filter, Membrane Solutions, https://www.membrane-solutions.com/contact_us.htm). All compounds were freshly prepared on the day of the experiment.

### 2.2. LMH Cell Culture

The chick liver hepatocellular carcinoma cell line LMH (CLS, Germany, lot no. 601411-714SF), from the 24th passage, was used in our experiments as a model cell line. Aspiration of odorous compounds acts first on the respiratory tract, but many findings suggest, that they are then transported with blood to other organs, and to the liver, which takes part in their detoxification. The LMH cells were cultured as a monolayer as previously described [10]. Briefly, they were cultured in collagen-coated T75 flasks in Waymouth’s Medium with 7.5% sodium bicarbonate (Gibco, Thermo Fisher Scientific, Waltham, MA, USA), 10% heat-inactivated foetal bovine serum (FBS) (Gibco, Thermo Fisher Scientific, Waltham, MA, USA), 25 mM HEPES (Sigma-Aldrich, St. Louis, MO, USA), and antibiotics (100 IU/mL penicillin and 100 µg/mL streptomycin, Sigma-Aldrich, St. Louis, MO, USA). The cells were incubated in a CO_2_ incubator (Galaxy 48S, New Brunswick, United Kingdom) at 37 °C in 5% CO_2_ for 7 days to reach 80% confluence. The confluent cells were detached with TrypLE^TM^ Express (Gibco, Thermo Fisher Scientific, Waltham, MA, USA) for 10 min at 37 °C, suspended in sterile PBS (Sigma-Aldrich, St. Louis, MO, USA), aspirated off the plastic flask, centrifuged (182× *g*, 5 min), decanted, and re-suspended in fresh medium. The cells were ready to use if they had a minimum of 90% viability as tested by Trypan blue exclusion.

### 2.3. Comet Assay (SCGE—Single Cell Gel Electrophoresis Assay)

The final concentration of LMH cells in each sample was adjusted to 10^5^ cells/mL. The cells in non-supplemented Waymouth’s Medium were incubated with specific concentrations of each odorous compound at 37 °C for 1 h. The final series of concentrations of the odorous compounds in culture were: 0.001–0.006% for DMA and ammonia, 0.001–0.12% for TMA, 0.003–0.5% for butyric acid and indole, and 0.0004–0.1% for phenol. The concentrations were selected based on our previous studies, in which we determined the IC_50_ values of these odorous compounds by MTT and PrestoBlue assays, which were 0.02–0.08% for ammonia; 0.03–0.06% for DMA; 0.02–0.08% for TMA; 0.11–0.32% for butyric acid; and, 0.06% for indole, depending on the incubation time and the assay [10]. The comet assay was performed under alkaline conditions (pH > 13), according to the procedure of Błasiak and Kowalik [11], as previously described [12]. In brief, after incubation, the cells were centrifuged (182× *g*, 15 min, 4 °C), decanted, suspended in 0.75% low melting point (LMP) agarose, layered onto slides pre-coated with 0.5% normal melting point (NMP) agarose, and lysed at 4 °C for 1 h in a buffer consisting of 2.5 M NaCl, 1% Triton X-100, 100 mM EDTA, and 10 mM Tris, pH 10. Next, the slides were placed in an electrophoresis unit, and DNA was allowed to unwind for 20 min in an electrophoretic solution consisting of 300 mM NaOH and 1 mM EDTA. Electrophoresis was conducted at 4 °C for 20 min at an electric field strength of 0.73 V/cm (300 mA). Finally, the slides were neutralised in distilled water, stained with 2.5 µg/mL propidium iodide (PI) (Sigma-Aldrich, St. Louis, MO, USA), and covered with cover slips. The slides were observed at 200× magnification under a fluorescence microscope (Nikon Eclipse Ci H600L, Tokyo, Japan) connected to a video camera and a personal computer-based image analysis system, namely Lucia-Comet version 7.0 (Laboratory Imaging, Prague, Czech Republic). About 50 to 100 images were randomly selected from each sample and the percentage of DNA in the comet tail was measured.

### 2.4. Lactate Dehydrogenase Activity (LDH) Assay

The LDH cytotoxicity assay is based on the leakage of a cytoplasmic enzyme called lactate dehydrogenase from cells when the plasma membrane is damaged. This assay is useful to detect necrosis [13].

In our experiment, 1 × 10^4^ LMH cells, in a complete culture medium, were placed in each well of a 96-well plate coated with collagen (BioCoat, Becton, Dickinson and Co., Franklin Lakes, NJ, USA). The cells were incubated for 24 h at 37 °C in 5% CO_2_ to allow them to attach to the collagen-coated surface. The following day, the medium was aspirated and 200 µL of each concentration of the test compound in Waymouth’s Medium without FBS, was added to each well in eight repeats. The control samples consisted of cells without any test agent. The cells were incubated in a CO_2_ incubator at 37 °C in 5% CO_2_ for 24 h in case of DMA, and 48h in case of ammonia and TMA. The final test concentrations were: 0.004–1.0% for DMA and ammonia, and 0.004–1.0% for TMA. The concentrations investigated, and the times of incubation were chosen to detect IC_50_ values. In our previous studies [10], the viability of the cells was measured by MTT and PrestoBlue assays. In this study, only the three most cytotoxic odorous compounds were chosen, namely ammonia, DMA and TMA, to check for the mechanism of cell damage (loss in cell membrane integrity and probably necrosis). The assay was conducted with a Cytotoxicity Detection Kit^PLUS^ (Roche, 04744934001, Basel, Switzerland), according to the manufacturer’s instructions. Three controls were included: background control (assay medium), low control (untreated cells), and high control (maximum LDH release). To determine the experimental absorbance values, the average absorbance values of the 8-repeat samples and controls were calculated and subtracted from the absorbance values of the background control. The cytotoxicity was determined as follows: cytotoxicity (%) = ((exp. Value-low control)/(high control-low control)) × 100. The absorbance was detected at 490 nm with the use of a microplate reader (TriStar^2^ LB 942, Berthold Technologies GmbH and Co. KG, Bad Wildbad, Germany). Results were presented as mean ± standard deviation (SD). The mean error of the method is up to 10%.

### 2.5. Calculation of IC_50_

The values of IC_50_, which is the concentration of the test compound required to reduce the cell survival rate to 50% of the control, were used as a degree of cellular sensitivity to a given treatment. IC_50_ values were determined according to the formula: IC_50_ = (X − Z)/(X − X_1_) × (C_X1_ − C_X_) + C_X_, where X is a 50% decrease in viability; X is % of viability > Z; X_1_ is % viability < Z; C_X_ is concentration of the compound for X, and C_X1_ is concentration of the compound for X_1_ [10,14].

### 2.6. Fluorescence Microscopic Analysis

The nuclear changes in LMH cells in the presence of test compounds were observed using 8-well Lab-Tek™ Chamber Slides (Nunc, Thermo Fisher Scientific, Waltham, MA, USA). Before culture, the slides were coated with collagen I (Gibco, Thermo Fisher Scientific, Waltham, MA, USA), according to the manufacturer’s instructions. LMH cells were seeded on to each well at a concentration of 2.5 × 10^5^ cells/well. In this experiment, three of the most cytotoxic odorous compounds were selected, which included ammonia, DMA, and TMA, to check for the mechanism of cell damage or apoptosis using DAPI (4’,6-diamidino-2-phenylindole) staining. The final test concentrations were 0.03% for ammonia, DMA, and TMA, which were close to the IC_50_ values estimated in MTT assay after 48 h incubation [10].

After exposure to these compounds, the medium with compounds was removed, cells were washed with PBS (phosphate buffer saline) (pH 7.2) and fixed with 80% ethanol (for 20 min at room temperature). After air-drying, the cells were stained with 1 μg/mL DAPI in the dark. The morphology of cells was observed at 1000× magnification under a fluorescent microscope (Nikon Eclipse Ci H600L, Tokyo, Japan), connected to a digital camera (Nikon Digital Sight DS-U3, Tokyo, Japan), and analysed using NIS-elements BR 3.0 imaging software (Nikon, Tokyo, Japan).

### 2.7. Statistical Analysis

Comet assay data were analysed using two-way analysis of variance (ANOVA), while a particular mode of interaction × time was used to compare the effects induced by the chemicals at this mode of interaction. Differences between samples with normal distribution were evaluated by Student’s *t*-test. Both Student’s *t*-test and ANOVA were performed using OriginPro 6.1 software (Northampton, MA, USA). Significant differences were accepted at *p* < 0.05. The results were presented as mean ± standard error of the mean (S.E.M.) (for the comet assays), and ± standard deviation (SD) (for LDH).

## 3. Results

### 3.1. DNA Damage in Chicken Liver Hepatocellular Carcinoma Cells

**Figure 1:**
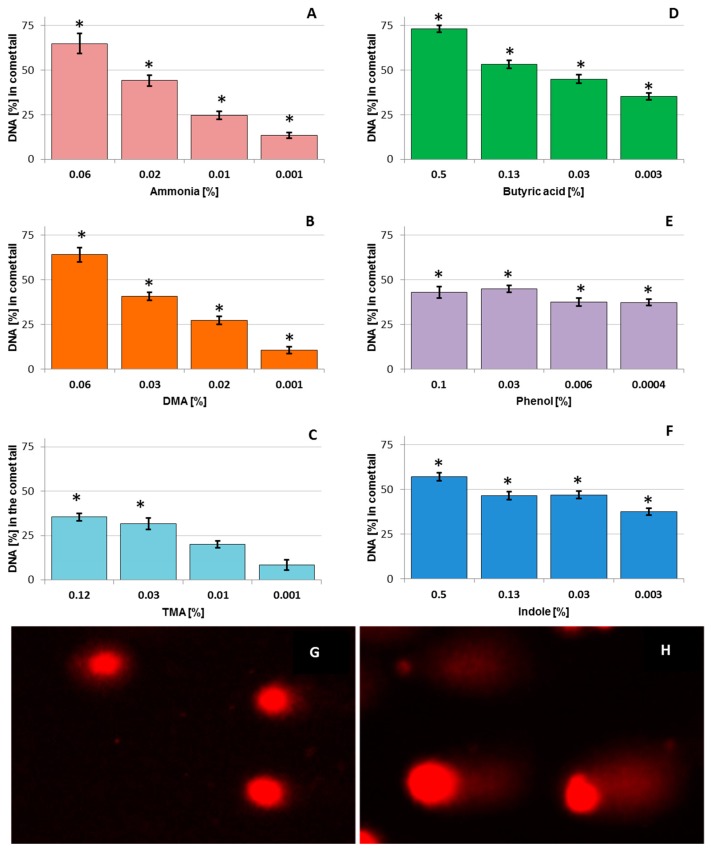
DNA damage in model chicken cell line (LMH) chicken cells after exposure to odorous compounds, such as (**A**) ammonia, (**B**) dimethylamine (DMA), (**C**) trimethylamine (TMA), (**D**) butyric acid, (**E**) indole and (**F**) phenol, expressed as the mean percentage of DNA in the comet tail in the alkaline comet assay. About 50 to 100 cells were analysed for each treatment. Data shown was obtained from two independent experiments. Error bars denote S.E.M. * Results were significantly different from unexposed control, ANOVA (*p* < 0.05). **G** and **H** show representative images of 2.5 µg/mL propidium iodide-stained comets: (**G**) untreated control and (**H**) sample exposed to 0.03% dimethylamine (extensive damage) in the alkaline comet assay.

Figure 1 displays the mean percentage of tail in the DNA of chicken hepatocytes that were exposed to odorous compounds and analysed by the alkaline comet assay. Representative images of control comets and actual sample after 0.03% DMA treatment, stained with propidium iodide are also shown (Figure 1).

Non-exposed cells (negative control) induced DNA damage of 5.0 ± 0.8%, while cell treatment with the positive control (20 μM H_2_O_2_) resulted in 42.6 ± 4.2% DNA breakage.

Ammonia, DMA, TMA, and butyric acid increased tail DNA in a dose-dependent manner at all concentrations. Ammonia and DMA at 0.06% concentration were highly genotoxic inducing 64.9 ± 5.6% and 64.0 ± 3.9% DNA damage, respectively (*p* < 0.05). 0.001% of ammonia, DMA, and TMA, induced mild and moderate genotoxicity in LMH cells of up to 13.3 ± 1.6%, 10.5 ± 1.6%, and 8.3 ± 2.9%, respectively. Butyric acid induced extensive DNA damage at all concentrations, reaching (73.2 ± 1.9)% at 0.5% concentration, and (35.2 ± 1.8)% at 0.003% concentration. In contrast, phenol and indole highly increased tail DNA independent of the concentration. The genotoxicity of phenol and indole fluctuated from (37.5 ± 1.9)% to (57.1 ± 2.2)%, and from (37.3 ± 1.8)% to (43.0 ± 3.3)%, respectively. Simultaneously, phenol and indole induced very extensive DNA damage, so the results on the graph do not reflect the actual effects. Higher doses of both these compounds resulted in complete DNA fragmentation in many cells, what can be the result from cell death, but not true DNA damage. Also, the number of comets per slide was lower than that for the lower concentrations and in unexposed cells. This indicates a strong cytotoxicity of these compounds on the cells after 1-h exposure.

### 3.2. Cytotoxicity and Determining IC_50_

We next investigated the mechanism by which odorous compounds can act on cells. Because butyric acid, phenol, and indole are formed in the farmhouses in low amounts [7,8,9], to the part of the research we chose the three main odorous compounds—ammonia, TMA, and DMA. We checked whether they affect cell membranes. One marker of membrane integrity is the level of the enzyme lactate dehydrogenase (LDH) that is released into the culture media. The LMH cells were challenged with the compounds for 24 or 48 h to observe the IC_50_ value, with concentrations ranging from 0.004% to 1.0% in eight repeats for each concentration.

**Figure 2:**
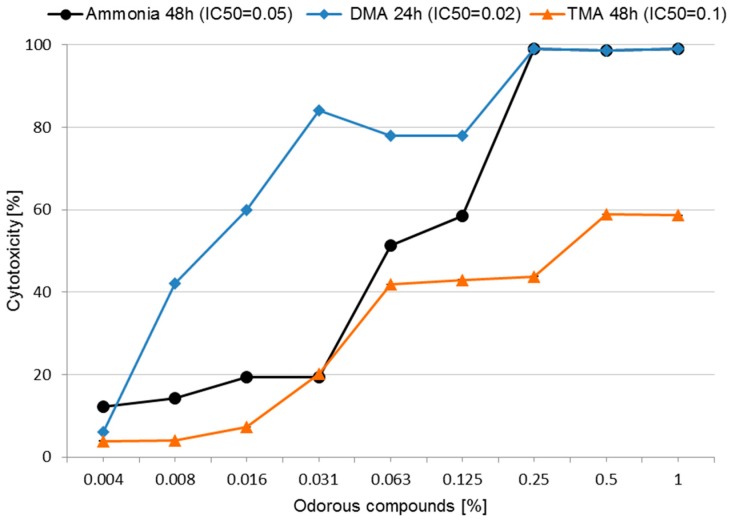
Cytotoxicity of ammonia, dimethylamine (DMA) and trimethylamine (TMA) in Lactate Dehydrogenase Activity (LDH) assay in LMH chicken cell line after 24 h (DMA) and 48 h (ammonia and TMA) exposure. Each data point represents the mean of the absorbance values of cells from eight individual wells (±SD). * *p* < 0.05 for concentrations of ammonia, DMA and TMA as compared to unexposed controls.

All odorous compounds caused a dose-dependent release of LDH. Exposure to 0.031% concentration of ammonia showed a significant (*p* < 0.05) increase in cytotoxicity, which reached nearly 100% at concentrations of 0.25%, 0.5%, and 1% (Figure 2). DMA had the highest cytotoxicity even in the presence of concentrations as low as 0.008% (42 ± 0.1)%, indicating the disruption of the cell membrane structure. TMA appeared to be the least cytotoxic in the LDH assay, with the release in LDH reaching a maximum of (58.6 ± 0.04)% at 1% concentration of TMA.

LMH cells treated with DMA resulted in high amounts of LDH release after only 24 h, while those treated with ammonia and TMA took 48h; hence the different times of incubation. The IC_50_ of DMA was calculated after 24 h of exposure to be 0.02%. For ammonia and TMA, IC_50_ could be determined only after 48 h of exposure, and was found to be 0.05% and 0.1%, respectively (Figure 2).

### 3.3. Nuclear Morphology of LMH Cells

**Figure 3:**
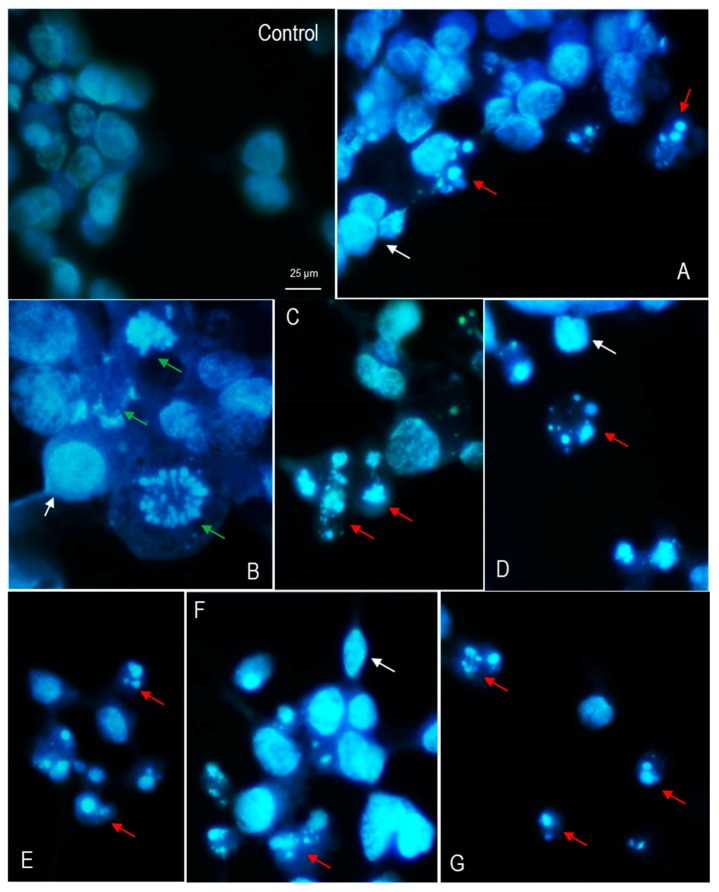
Nuclear morphology of LMH cells stained with DAPI (4′,6-diamidino-2-phenylindole) after 48h exposure to 0.03% ammonia (**A**–**C**), dimethylamine (**D**,**G**) and trimethylamine (**E**,**F**). Condensation of nuclear material (white arrows), apoptotic bodies (red arrows), cell swelling and chromatin lysis (green arrows) were observed. Fluorescence microscopy (Nikon, Tokyo, Japan); 1000× magnification. Images are representative of two independent experiments.

Morphological alterations in LMH chicken cells after exposure to odorous compounds were qualitatively investigated using a fluorescent microscope. In the presence of ammonia (0.03%) (Figure 3A,C) chromatin condensation, cell shrinkage, as well as nuclear fragmentation (apoptotic bodies) were observed. Additionally, chromatin lysis and swelling of the cells were observed (Figure 3B), and this could possibly be due to necrosis. Chromatin condensation, shrinking of the cells, nuclear margination, and apoptotic bodies appeared after exposure to 0.03% DMA (Figure 3D,G) and TMA (Figure 3E,F).

## 4. Discussion

Odorous compounds generated from chicken sheds are a potential nuisance to the environment and health of humans and animals. There are insufficient in vitro studies investigating geno- and cytotoxic mechanisms of poultry odorous compounds. We applied the **comet assay** to determine the mechanisms of action of the test odorous compounds. The assay is able to detect even low levels of DNA damage in a cell [15]. It can detect a wide range of DNA damage, such as single and double-strand breaks, base damage, inter-strand or DNA-protein cross-links, alkali labile sites, oxidised purines, and pyrimidines, as well as extensive DNA fragmentation [16]. The test concentrations should cover a range from the maximum acceptable cytotoxicity to little or no cytotoxicity, because DNA damage is associated with cell death [15]. In the current study, we selected concentrations based on our previous research, in which we investigated the cytotoxic activity of test odorous compounds using the MTT assay [10]. In the alkaline comet assay, all odorous compounds induced heavy damage to DNA in LMH cells. Butyric acid, phenol, and indole, triggered the most extensive genotoxic effects to the cells, as much as 35%; in low concentrations, and for indole and phenol, it was also at a similar level. This is evidence of their high cytotoxicity to cells in suspension and inhibition of the DNA repair system (repair system has been overwhelmed by extensive damage). In contrast, ammonia, DMA, or TMA induced considerable damage to DNA of up to 64% at higher concentrations, and the results were comparable to the dose.

When treated with a toxic compound, living cells may stop growing and dividing, or die through necrosis or apoptosis. Generally, cells undergoing necrosis swell and loose membrane integrity, following which they shut down and release their intracellular contents into the extracellular area. In contrast, cells undergoing apoptosis are characterised by the shrinking of the cytoplasm, chromatin condensation, and DNA fragmentation [13]. Lactate dehydrogenase is an enzyme found inside every living cell. In **LDH leakage assay**, a tetrazolium salt is used in this test. When cell membrane integrity is compromised, the presence of this enzyme in the culture medium can be used as a cell death indicator. In the first stage, lactate dehydrogenase produces reduced NADH (nicotinamide adenine dinucleotide) when it catalyses the oxidation of lactate to pyruvate. In the second stage, a tetrazolium salt is converted to a coloured formazan using newly synthesized NADH in the presence of an electron acceptor. The amount of formazan can be quantified by standard spectroscopy. The assay enumerates the percentage of necrotic cells in a sample. Unfortunately, LDH release assay does not distinguish between primary necrosis and secondary necrosis as a consequence of apoptosis [13]. In the current research, the most cytotoxic compound in LDH assay was DMA, for which the IC_50_ (0.02%) was estimated just after 24 h incubation. Ammonia also showed high cytotoxicity, but it was only possible to estimate IC_50_ after 48 h (0.05%). The least cytotoxic was TMA (IC_50_ calculated after 48 h was 0.1%). Similar results for ammonia, DMA, and TMA were achieved in our previous study [10] using the MTT (3-[4,5-dimethylthiazol-2-yl]-2,5-diphenyltetrazolium bromide) and PrestoBlue assays. These three compounds seem to be more toxic in the MTT assay. These differences highlight the differences in the mechanisms of cytotoxicity of these odorous compounds, and the different nature of each test. The LDH leakage assay is based on the release of the enzyme into the culture medium after cell membrane damage (cell death), while the MTT assay is based on the enzymatic conversion of MTT in the mitochondria. The formazan product is impermeable to the cell membranes, and therefore, it accumulates in healthy cells [17]. Thus, we can conclude, that the odorous compounds are destructive for mitochondria.

**DAPI staining** delivers a rapid and convenient assay for apoptosis based on fluorescent detection. DAPI is a specific DNA binding dye. This dye is not completely permeable, and normal cells give slight blue fluorescence with the round nucleus stained uniformly with its clear margins. Along with the apoptosis process, the ability of permeability improves, and the apoptotic cells produce high blue fluorescence, the margination of the nucleus is observed, and the condensation of chromatin is easily stained. Cell shrinkage, chromatin condensation, nuclear fragmentation, and nucleus margination are associated with the apoptotic mode of cell death. In our research, the cytotoxic effect of the test odorous compounds was supported by microscopic observations of nuclear morphological changes. Cells incubated for 48 h with 0.03% ammonia presented nuclei with either apoptotic (apoptotic bodies), or necrotic morphology, with nucleus fragmentation of different sizes. Some nuclei appeared to be dispersed within the cytoplasm, and cell swelling was observed. Apoptotic symptoms dominated after treatment with 0.03% DMA or TMA. In our previous study [10], by using Giemsa/May-Grünwald staining we demonstrated that the treatment of LMH cells with these compounds produced *inter alia* chromatin condensation and fragmentation. The IC_50_ doses of test odorous compounds changed nuclear morphology and induced apoptosis in LMH cells, but the data requires confirmation by other tests.

Genotoxic activity of **ammonia** was documented by several researchers. In this paper, using the comet assay we found that, depending on the concentration, ammonia induced extensive DNA damage in chicken cells. In contrast, Yadav and Kaushik [18] found that ammonia induced micronuclei formation in Swiss albino mice, and inhibited DNA repair in mouse fibroblasts. One study examined the genotoxic effect of ammonia in humans [18]. Analysis of blood samples, from 22 individuals exposed to ammonia (working in a fertilizer factory), and 42 control individuals not exposed to ammonia, showed increased frequency of chromosomal aberrations, sister chromatid exchanges, and an increased mitotic index in blood samples of exposed individuals. The frequency of DNA damage increased along with the increasing length of exposure. In our research, geno- and cytotoxic activity of ammonia could occur as a result of alkalisation. But Mouille et al. [19] demonstrated that ammonia acts on colon HT-29 cells as an antimitotic agent, independent of the pH. We showed that ammonia changes the morphology of the nuclei in LMH chicken cells. Mouille et al. [19], using in vivo animal experiments with ammonia, showed that excessive amounts of odorous compounds alter colonic epithelial cell morphology and increase cell proliferation. Contrary to our findings, the authors established that ammonia (20 mM NH_4_Cl) did not significantly affect the viability of human colon HT-29 cells by decreasing the integrity of the cell membrane (LDH release). Our findings support geno- and cytotoxic action of ammonia, what was proved by other researches, with the application of another or the same methods, but different models of the cell lines. Pan et al. [20] examined the toxicity of ammonia present in urea and chicken manure, on plant roots, including wheat, canola, and faba, which developed expanded toxicity zones initiated at the root apex. The authors observed progressive necrosis and shrinking of the root axis and root hairs. Ammonia sources also caused inhibition of root elongation within 4 to 8 h of exposure. Gupta et al. [21] observed the toxic effects of poultry litter aqueous leachate in *Ceriodaphnia dubia*, and the main constituents of the leachate was ammonia and anionic organic compounds. Toxicity of water elutriates of chicken manure in protozoa, such as *Paramecium caudatum*, as well as the crustacean *Daphnia magna*, as test objects was demonstrated by Galitskaya and Selivanovskaya [22]. Delgado et al. [23] showed phytotoxicity of poultry manure in a study using the cress. Poultry litter leachate was found to be mutagenic to *Salmonella* Typhimurium strains TA 97, TA 98, TA 100, and TA 102 using the Ames test [24]. It is necessary to mention that ammonia accumulates as a result of glutamine metabolism, and is a toxic by-product of animal cell culture [25,26]. It can exhibit such negative effects as inhibition of cell growth, protein production and glycosylation [26]. Following DAPI staining of LMH cells after exposure to ammonia, characteristic apoptosis symptoms were observed. Mirabet et al. [27] demonstrated that ammonia can induce cell death due to apoptosis, and that the toxic effects caused by ammonia are different depending upon the cell line type.

In the comet assay we showed that **DMA** and **TMA** increased DNA damage in a dose-dependent manner. Genotoxic activity of DMA was studied in *Saccharomyces cerevisiae* D7 and evaluated by Galli et al. [28]. DMA induced conversion of gene and point reverse mutation after metabolic activation. It also showed hepatotoxic effect by a significant reduction in the activity of selected monooxygenase enzymes. Pool et al. [29] showed that DMA induced single-strand DNA breaks in hepatocytes of rats, hamsters, and pigs using a 1 or 3-h culture suspension technique. The breaks were seen in liver cells after treatment with 1 mg/kg DMA, and in kidney and lung cells at a dosage of 20 mg/kg DMA [30]. **TMA** appeared to be genotoxic/mutagenic in Ames test with *Salmonella* Typhimurium [31]. It also induced chromosomal aberrations without metabolic activation in the Chinese hamster cell line [32,33]. The authors proposed that the mechanism of its action was a shift in pH. In our study, the test samples were not neutralized. Therefore; the mechanism of their geno- and cytotoxic action may have been related to a pH shift.

We demonstrated that **phenol** and **indole** induced strong DNA damage, independent of the concentration. **Phenol** was assessed as a genotoxin by Li et al. [34]. The authors showed that phenol caused a significant increase in micronucleus frequencies when compared to the solvent control. It also induced statistically significant increases in DNA damage (in comet assay) on three different biomaterials (i.e., lymphocyte of human, spermatid of mouse, and akaryocyte of crucian). Phenol appeared to be mutagenic in vitro in *Vicia faba* and mouse spleen cells (sister chromatid exchange) [35], induced DNA oxidative damage in human promyelocytic HL-60, and mouse bone-marrow cells [36]. Phenol also induced sister chromatid exchange in human lymphocytes [37,38]. It also showed genotoxicity in a micronucleus test in mouse bone-marrow cells [39,40]. **Indole** and its derivatives are derived from bacterial degradation of tryptophan, and contributes to the characteristic odour of faeces. Reddy et al. [41] evaluated the genotoxic potential of indole derivatives (3-methylindole, melatonin, serotonin, and tryptamine) in vitro. All compounds produced DNA adducts in calf thymus DNA in the presence of rat liver S9. In cultured rat hepatocytes, all produced DNA adducts. In our research, **butyric acid** induced extensive DNA damage. The ability of **butyric acid** to inhibit cell growth, followed by the increase in apoptosis in Jurkat human T lymphocytes was described by Kurita-Ochiai et al. [42]. The authors indicated that butyric acid has bimodal effects on cell proliferation and survival. The apoptosis was induced by high levels of butyric acid. Several other studies have also demonstrated the ability of butyric acid to induce apoptosis in different cell lines in vitro [43,44,45].

In our study, low concentrations of ammonia and TMA showed little or no cytotoxicity in the LDH assay, but were found to be highly genotoxic in the comet assay. The same effect as in the LDH assay was observed for all tested odorous compounds in the MTT assay (in our previous study) [10]. It arises from the fact that a cytotoxic agent may be genotoxic (cause damage to DNA), or may be destructive for cell organelles (e.g., cell membrane or mitochondria), but not each cytotoxic chemical must induce genotoxicity. Furthermore, genotoxic substances may induce DNA damage at non-cytotoxic concentrations. Genotoxicity, may lead to cancer-causing mutations, but it does not have to be cytotoxic for the cell. From our research we can conclude, that ammonia, DMA, and TMA are very harmful to LMH cells, probably because of some similarities in their chemical structures, and hence cellular interactions leading to downstream toxicity.

## 5. Conclusions

Our findings suggest, that odorous compounds can be cyto- and genotoxic in vitro, hence they are also likely to be harmful in vivo. Ammonia, DMA, TMA, phenol, indole, and butyric acid have extensive geno- and cytotoxicity, which could be caused by changes in the pH of the medium. Ammonia, DMA and TMA induce cytotoxicity in LMH chicken cell line by loss in cell membrane integrity and, probably, necrosis. They also change the nuclear morphology of LMH cells, causing chromatin condensation and fragmentation, and also cause an increase in apoptotic bodies, as observed with DAPI staining. Thus, these compounds can induce cell death in two ways: necrosis and apoptosis, but the findings need to be confirmed using additional in vitro studies involving tests for detecting apoptosis. Finally, our research demonstrates that the comet and LDH assays, as well as DAPI staining, are appropriate and sensitive methods to screen selected odorous compounds for geno- and cytotoxicity testing.
